# Some essential pre-requisites and requirements to establish and run a successful Journal

**DOI:** 10.12669/pjms.40.4.9408

**Published:** 2024

**Authors:** Shaukat Ali Jawaid

Numerous medical universities, medical and dental colleges are currently venturing into publishing their scientific journals. Moreover, several professional specialty organizations aspire to initiate their own journals. However, it’s crucial for these entities to recognize that ensuring a journal’s long-term sustainability necessitates specific prerequisites and essential criteria. Primarily, establishing a successful journal requires teamwork—no individual can accomplish this task alone. In reality, publishing a journal often becomes a highly stressful and demanding endeavor. 1-3

Acquiring authorization (Declaration) to publish a journal presents a significant challenge. The bureaucratic pace of government departments may elongate this process, stretching from one year to even longer. Once this initial hurdle is crossed, assembling a multidisciplinary Editorial Advisory Board is essential, favoring young, astute faculty members and committed healthcare professionals who can devote time to this academic pursuit. The selection of a unique journal title holds paramount importance. Instead of imitating established journals, opting for a distinctive title that can carve its own identity and become a recognizable brand in the future is crucial.

Setting up a functional office with essential staff—such as a Managing or Executive Editor, a computer operator, and an office assistant—constitutes the minimum human resource support for the Editor. Subsequently, focus on the following:

1. In the nascent stages, soliciting manuscripts for a newly established journal requires concerted efforts—reaching out to friends and well-wishers to submit their work. It’s important to adhere to the limits set by the Higher Education Commission and PM&DC regarding the percentage of papers the journal can publish from its own faculty members if the journal is owned and published by any medical institution.
2. Commencing with a biannual or quarterly publication, depending on resource availability, can be a prudent starting point, requiring a lesser number of initial manuscripts.
3. Ensuring a diverse range of content, encompassing original articles, Reviews, Clinical Case series, Case reports, special and brief communications, systematic reviews, conference proceedings, Editorials, and Guest Editorials, fosters greater readership. Regulatory bodies in Pakistan stipulate minimum numbers of original papers for recognition.
4. Designing a user-friendly website for the journal and utilizing various communication channels—social media, conferences, emails—to invite manuscripts enhances its visibility and, consequently, author submissions.
5. Establishing and regularly updating a Reviewers Database, engaging subject experts for manuscript reviews from the outset, and choosing an appropriate peer review system (single blind, double blind, or Open Peer Review) are critical decisions, each with its own advantages and disadvantages.
6. After publishing initial issues, seeking recognition from various indexing services and databases like the Directory of Open Access Journals (DOAJ),^4^ Scopus,^5^ and eventually applying for Clarivate indexing service (known for Impact Factor), Medline, and PubMed Central becomes pertinent. Prof. Akhtar Sherin in a recent editorial has given some useful information regarding international indexation. 6
7. Implementing author-friendly and reviewer-friendly policies, finalizing Editorial and Advertising policies, and selecting a preferred business model are essential steps.
8. Maintaining timely communication with authors, updating them on manuscript progress, and sending reminders for responses in case of delays are crucial for smooth operations.
9. Utilizing the Open Journal System for manuscript management offers numerous benefits and aligns with regulatory preferences.
10. Providing staff training and offering timely feedback to authors are integral components of journal management.
11. Each manuscript published must contain Ethics Committee/IRB approval with Reference Number and Date in the Methods Section. Retrospective studies can obtain waivers from the institutional EC/IRB approval. These alongwith peer review reports must be kept for at least two years as the regulatory bodies might ask for them.
12. In case the data is collected from different institutions, apart from EC approval permission to use the data from all the institutions is essential.
13. Ensure that all clinical trials accepted for publication are registered particularly the Randomized Clinical Trials (RCTs).
14. Trials can be registered with any registry national or international. DRAP in Pakistan has started a Trial Registry^9^ while many other countries and WHO also has trials registry.
15. Individual author’s contribution of all listed authors, the name of who is responsible for integrity of the research work must be clearly stated.
16. Other important requirements include Limitations of the study, Acknowledgements, conflict of interest and funding sources must be mentioned in each manuscript.
17. Authors instructions should clearly state that all manuscripts at the time of submission must be accompanied by Letter of Undertaking ensuring exclusive submission and signed by all listed authors, Ethics Committee/IRB approval and processing fee if applicable.
18. PDF file must be sent to the authors for proof reading before publication to ensure there are no mistakes left.
19. Ensure author must submit good quality illustrations, figures for good results. They must be uploaded on the website separately. Tables must be in word file so that they can be edited if need be. The whole manuscript with tables, figures, illustrations should be uploaded as one file first and tables, figures should be uploaded separately as well.
20. Some journals do not entertain manuscripts which are already published as preprints on different websites.
21. Journal must ensure uniformity in structured abstracts and formatting of the manuscripts. In references, use approved abbreviations of the Journals names, list up to six authors and then write et al. Add doi number if available.
22. In case of papers from Dissertations, apart from approval from RTMC of CPSP, ethics committee approval will also be required.
23. Time line of publication should be ensured. Details of date on which paper was submitted, date on which revision was received, final acceptance date must be mentioned prominently with each manuscript.
24. In case of Review articles make sure the authors follow latest ICMJE guidelines which state that details regarding the databases searched and the time period must be mentioned in the summary as well as in the methods section in the manuscript.
25. Journals Evaluation Committee of Paksitan Medical & Dental Council takes care of all these aspects and if the editors follow the above guidelines, they won’t face much problem with recognition.

University of Health Sciences (UHS) Lahore in collaboration with Paksitan Associate of Medical Editors (PAME) have started a few training courses for the editors.^10^ An Online CMEJ Course offered by CorTeach in collbration with King Edward Medical University Lahore is also avaliable. Make use of these facilities available for the professional capacity building of the Editor and other Editorial Staff members which will help in improving the standard of the journal.

## References

[1] Jawaid SA (2004). Problems faced by editors of peer reviewed medical journals. Saudi Med J.

[2] Jawaid SA, Jawaid M (2019). Common reasons for not accepting manuscripts for further processing after editor's triage and initial screening. Pak J Med Sci.

[3] Jawaid SA, Jawaid M (2013). Are the Editors faced with e-problems performing their duties and responsibilities satisfactorily?. Pak J Med Sci.

[5] https://www.scopus.com/home.uri.

[6] Akhtar Sherin The Road less Travelled:Nurturing a Medical Journal's Quest for International Indexation and Impact Factor. Annals of King Edward Med University -2023.

[7] Jawaid SA (2014). Authors- the most dangerous pressure group. Pak J Med Sci.

[8] https://www.icmje.org/.

[9] www.https://ctr.dra.gov.pk/.

[10] http://111.68.105.24/downloads/cmeamje.pdf.

